# The impact of early pregnancy metabolic disorders on pregnancy outcome and the specific mechanism

**DOI:** 10.1186/s40001-023-01161-z

**Published:** 2023-06-24

**Authors:** Xi-Zi Zhu, Zhi-Min Deng, Fang-Fang Dai, Hua Liu, Yan-Xiang Cheng

**Affiliations:** Department of Obstetrics and Gynecology, Renmin Hospital of Wuhan University, 99 Zhang Zhidong Road, Wuhan, 430060 Hubei China

**Keywords:** Miscarriage, Glucose metabolism, Lipid metabolism, Amino acid metabolism, Redox reactions

## Abstract

Miscarriage is the most common complication of pregnancy. The most common causes of early miscarriage are chromosomal abnormalities of the embryo, maternal endocrine abnormalities, organ malformations, and abnormal immune factors. Late miscarriages are mostly caused by factors such as cervical insufficiency. However, the causes of 50% of miscarriages remain unknown. Recently, increasing attention has been given to the role of metabolic abnormalities in miscarriage. In this review, we mainly discuss the roles of four major metabolic pathways (glucose, lipid, and amino acid metabolism, and oxidation‒reduction balance) in miscarriage and the metabolism-related genes that lead to metabolic disorders in miscarriage. Depending on aetiology, the current treatments for miscarriage include hormonal and immunological drugs, as well as surgery, while there are few therapies for metabolism. Therefore, we also summarize the drugs for metabolism-related targets. The study of altered metabolism underlying miscarriage not only helps us to understand the mechanisms involved in miscarriage but also provides an important basis for clinical research on new therapies.

## Introduction

The most common complication of pregnancy is miscarriage, defined as spontaneous abortion of the foetus within 28 weeks of gestational age. The European Society of Human Reproduction and Embryology (ESHRE) defined recurrent spontaneous abortion (RSA) as the loss of two or more pregnancies with the same sexual partner [1]. It is estimated that approximately 20% of pregnancies end with miscarriage (< 20 weeks), and the average prevalence of miscarriage in females is 11% [2, 3]. Clinical miscarriages can be subdivided into early (before 12 weeks of pregnancy) and late clinical miscarriages (12 to 28 weeks of pregnancy). Previous studies have suggested many different causes of miscarriage, including anatomical, genetic, endocrine, and immunological disorders, as well as various infections and environmental factors. Nonetheless, the potential factors remain obscure in roughly half of situations for which the aetiology is unclear at this point [4–6]. Therefore, there is an urgent need to determine the mechanisms of miscarriage to clearly understand its causes.

In recent years, a developing number of studies have demonstrated that metabolism is altered during pregnancy and has a significant impact on pregnancy outcomes. In one study, metabolomic analysis of maternal blood samples distinguished 4995 metabolic profiles (9651 in total), 460 annotated compounds (687 in total), and 34 human metabolic pathways (48 in total) that were fundamentally modified during pregnancy [7]. Li et al. [8] recognized 54 potential metabolites utilizing gas chromatography–time-of-flight mass spectrometry (GC-TOFMS) and distinguished glycine, serine, threonine, β-alanine, the tricarboxylic acid (TCA) cycle, and phenylalanine metabolism as key biological pathways in the development of RSA. With advances in research on miscarriage and metabolism-related studies, there is expanding mindfulness that abnormalities in metabolites and metabolic pathways are common hallmarks in patients with RSA; however, treatment focusing on metabolic pathways has not yet become a therapeutic objective. Here, we first reviewed the changes in four metabolic pathways during normal pregnancy. Then, we explored the mechanisms by which metabolic abnormalities lead to miscarriage, along with the medications used against these targets, and eventually summarized the metabolism-related genes leading to miscarriage.

## Metabolic alterations in normal pregnancy

During pregnancy, the mother experiences many metabolic adaptations to provide her with sufficient energy stores to meet the demands of pregnancy. During the first 6 months of pregnancy, the mother is in an anabolic period characterized by hyperphagia, increased insulin sensitivity, and lipid stores [9, 10]. Conversely, the last trimester of pregnancy is portrayed by a catabolic state, hyperinsulinaemia, diminished insulin responsiveness, and increased placental exchange of supplements [11, 12]. These adaptations help to provide the appropriate environment for normal foetal growth in the uterus and prepare the mother for breastfeeding [13, 14].

In this review, we discuss the four major metabolic pathways associated with pregnancy and how their abnormalities result in miscarriage, including glucose, lipid, and amino acid metabolism, and oxidation‒reduction balance. In addition, we also summarize the medications for the relevant targets as well as the metabolism-related genes that contribute to adverse pregnancy outcomes. This will not only deepen our understanding of the mechanisms of miscarriage but also provide the theoretical foundation for clinical explorations of new treatments.

### Glucose metabolism

In early gestation, the embryo depends on glycolysis to produce adenosine triphosphate (ATP), and the most important source of the required substrates is glycogen stored in the endometrium [15]. To meet the increased metabolic needs, glucose homeostasis during pregnancy is altered, especially by the transient state of insulin resistance, which is compensated by the proliferation of pancreatic beta cells and the increasing insulin secretion capacity stimulated by glucose [16–18]. On one hand, prolactin, as a known regulator of beta-cell growth and function, acts at multiple scales to prepare the mother for the new demands associated with the offspring. As pituitary prolactin secretion ceases, serum concentrations of leptin, oestradiol, progesterone, and other placental hormones increase with gestation, and these hormones also work together to maintain insulin resistance during pregnancy [19, 20]. On the other hand, due to the high substrate demand and the inefficiency of foetal gluconeogenesis, the fetoplacental unit forms a system for the rapid transfer of glucose from the maternal blood to the placenta. This transfer system is mainly associated with the expression of glucose transporter proteins (GLUTs), which promote insulin responsiveness [21]. There are six GLUTs confirmed in the placenta to date: GLUT1, 3, 4, 8, 9, and 12, with GLUT1, 3, and 4 assuming a significant role [22, 23]. The characteristics and regulatory factors of selected GLUTs in human placenta have been described in detail in the review by *Stanirowski PJ *et al*. *[24]. The expression of GLUT1 increased with the progression of pregnancy [25], whereas the expression of GLUT3 was significantly higher in hypoxic conditions in early gestation and diminished in late gestation [26, 27]. In general, the maternal body is regulated by several hormones to maintain the state of insulin resistance during pregnancy, while increased insulin secretion promotes the expression of GLUT in the foetal placental unit to provide adequate glucose and energy for the foetus.

### Lipid metabolism

Changes in maternal lipid metabolism during human pregnancy may be classified according to the anabolic and catabolic periods. Of these, the anabolic period occurs in the initial six months of human pregnancy and is dominated by increasing lipid deposition in maternal tissues [10, 12, 28]. At the macroscopic scale of lipid synthesis, during this period, the mother usually increases her food intake and the accumulation of body fat. At the molecular scale, research has shown that in rats, the conversion of glucose to fatty acids and glycerin gradually increases by the 20th day of gestation [29]. In addition, the activity of lipoprotein lipase (LPL) was enhanced in plasma; in contrast, its activity in adipose tissue did not change significantly. The functions of LPL differ between the two locations. In plasma, LPL can promote the absorption of lipids by hydrolysing triglyceride-rich chylomicrons and very low-density lipoproteins [14, 30], while LPL in adipose tissue contributes to the deposition of fat [31]. In late pregnancy, multiple hormones, including insulin, progesterone, cortisol, prolactin, oestrogen, and leptin [32, 33], mediate increased mRNA expression and the activity of hormone-sensitive LPL in white adipose tissue, thereby promoting lipolysis [34]. Thus, maternal lipid synthesis and accumulation increase in early gestation (increased intake, increased gluconeogenesis, increased plasma LPL activity), with little change in catabolism (little change in LPL activity in adipose tissue), while catabolism increases in late gestation (promoting LPL activity in adipose tissue). In contrast, the mother promotes LPL activity in adipose tissue in late pregnancy through the combination of multiple hormones, which increases lipolysis.

### Amino acid metabolism

The maternal demand for amino acids increases during pregnancy for the establishment of the metabolic microenvironment of the endometrium in preparation for implantation and early pregnancy. Increased amino acid metabolism provides not only required proteins for exponential foetal growth, but also intermediate metabolites that promote multiple biosynthetic pathways [35]. Most amino acids are found at higher concentrations in foetal plasma than in maternal plasma, suggesting their active accumulation in the syncytial trophectoderm [36, 37]. The active uptake of neutral amino acids by syncytiotrophoblast cells is mainly mediated by the combination of sodium-coupled neutral amino acid transporters (SNATs) and non-sodium-dependent L-type amino transporters (LATs). The expression and activity of SNAT, the A system, increases with gestational age and foetal size [38, 39]. Meanwhile, the LAT, or L system, is present in only a few normal tissues and is primarily responsible for the transport of essential amino acids [40]. As a consequence, circulating amino acid concentrations increase during pregnancy and are actively transported into foetal tissues through special placental amino acid transporters (the A and L systems), which provide amino acids for foetal growth and development.

### Oxidation–reduction balance

In the female reproductive system, physiological levels of reactive oxygen species (ROS) serve an important regulatory role through various signal transduction pathways in folliculogenesis, oocyte maturation, the endometrial cycle, embryogenesis, and pregnancy [41]. Under normal physiological conditions of the placenta, the balance between the elements of the intrinsic redox reaction is maintained mainly through antioxidant reactions mediated by the Keap1-nuclear factor-erythroid 2 related factor 2 (Nrf2) pathway [42–44]. Furthermore, in the hypoxic environment of the placenta during gestation, the hydroxylation of hypoxia-inducible factor-1α (HIF-1α) (as its common degradation pathway) decreases, leading to HIF-1α accumulation and nuclear translocation [45]. HIF-1α promotes the transcription of the forkhead box protein P3 (FoxP3) gene and the production of regulatory T cells under hypoxic conditions, which promotes immune tolerance and reduces oxidative stress [46, 47]. In summary, the balance between ROS and antioxidants during pregnancy is essential and is mainly maintained by Keap1/Nrf2 and FoxP3 activated by HIF-1α in the specific hypoxic environment of the placenta.

## Abnormal metabolism and miscarriage

### Abnormal glucose metabolism and miscarriage

A previous study found that women with recurrent miscarriages are more likely to have abnormal glucose metabolism [48]. In the following, we detail the studies related to abnormal glucose metabolism and abortion through three aspects: (i) glycogen synthesis; (ii) glycolysis and HIF; and (iii) CD39- and CD73-mediated ATP metabolism.

In terms of glycogen synthesis, data show that glycogen accumulates tenfold to meet the energy metabolic substrate requirements during pregnancy, and this process primarily relies on GLUT or sodium-glucose cotransporter (SGLT) [49, 50]. Studies have proven that SGLT1 gene and protein expression is significantly reduced in the endometrium of RSA patients during the implantation window [51, 52]. This was corroborated in the SGLT1-deficient mouse model, in which endometrial glycogen, litter size, and pup birth weight were lower than those of wild-type mice. This leads us to conclude that SGLT1 deficiency in the human endometrium at implantation can lead to miscarriage and intrauterine growth restriction through decreased glycogen synthesis.

It has been relatively well demonstrated that HIF-1α is involved in the regulation of glucose metabolism homeostasis under hypoxic conditions. It acts as an oxygen-sensitive transcriptional activator and can induce the transcription of a variety of genes related to gluconeogenesis [53]. Lactate (LA) is an important metabolite in hypoxia-inducible factor (HIF)-mediated glycolysis and is synthesized by lactate dehydrogenase A (LDHA) upon activation by highly expressed HIF-1α under hypoxic conditions [54]. Lactate can act as an active metabolite in physiological, immunological, and cell-biological regulation through the mediation of the monocarboxylate transporter protein (MCT) [55–57]. It has been shown that LA content is significantly elevated in the decidua of RSA patients. LA enhances inducible nitric oxide synthase (INOS) expression in a HIF-1α-dependent manner, which in turn promotes M1 polarization of decidual macrophages, leading to the disruption of immune tolerance to trigger miscarriages [58]. The studies of mouse miscarriage models in this manuscript have also shown that blocking LA uptake with AZD3965 (MCT-1 inhibitor) could improve pregnancy outcomes, suggesting that MCT-1 could be a potential therapeutic target for RSA. Since the HIF family plays an important role in regulating glucose metabolism homeostasis, its degradation pathways are of equal interest [59, 60]. It was found that a decrease in chorionic succinate, an intermediate product of the tricarboxylic acid cycle during pregnancy, could promote HIF-1α degradation via the PHD-VHL pathway by promoting the hydroxylation of HIF-1α [61]. This leads to a decrease in HIF-1α, which inhibits angiogenesis, invasive migration of trophoblast cells and glycolysis and ultimately causes RSA. It is evident that while HIF regulates glucose metabolism homeostasis, the glycolytic products LA and succinate can also influence pregnancy outcomes by affecting HIF levels.

Abnormalities in the metabolism of ATP, the product of glucose metabolism, which is the direct provider of energy during pregnancy, are also closely associated with miscarriage. The ATP adenosine metabolic pathway modulated by CD39/CD73 has recently been suggested to play a significant role in immunosuppression [62]. CD39 hydrolyses ATP and ADP to produce AMP, and the membrane-bound 5’-nucleotidase CD73 further hydrolyses AMP into adenosine [63]. Therefore, CD39 and CD73 can convert proinflammatory immune cells driven by ATP to anti-inflammatory immune cells evoked by adenosine, thus causing immunosuppression [64, 65]. It was shown that downregulation of the TGF-β/mTOR/HIF-1α pathway leads to the inhibition of ATP-adenosine metabolism and causes a decrease in the number of CD39^+^ and CD73^+^ cells at the maternal–foetal interface [66]. This depresses the proliferation and invasion of trophoblast cells, reduces apoptosis and increases the cytotoxicity of decidual natural killer (dNK) cells, which contributes to RSA.

**Fig. 1:**
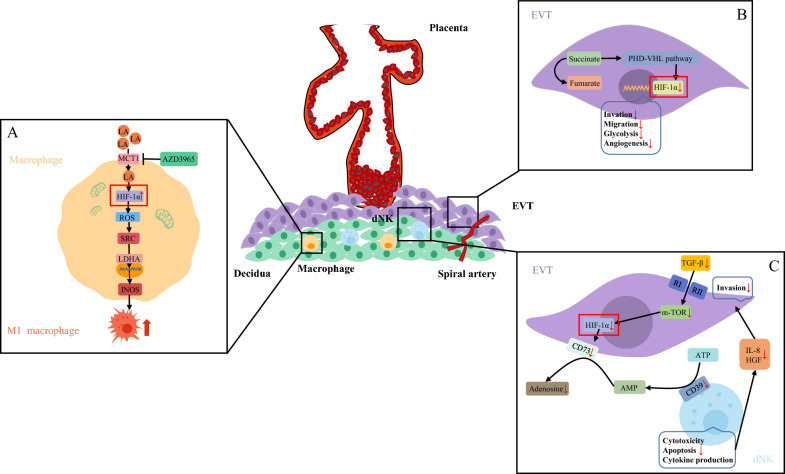
The role of HIF-1α in abnormal glucose metabolism leading to abortion. **A**. Elevated lactate levels in metaphase macrophages activate the HIF-1α/SRC/LDHA pathway, enhancing INOS expression in a HIF-1α-dependent manner, which in turn promotes their M1 polarization, thereby disrupting immune tolerance and triggering abortion. AZD3965 can reverse. **B**. Insufficient accumulation of chorionic succinate promotes HIF-1α degradation via the PHD-VHL pathway, leading to a decrease in HIF-1α and thereby inhibiting angiogenesis, trophoblast migration, and glycolysis. **C**. Downregulation of the TGF-β/mTOR/HTF-1α pathway leads to inhibition of ATP-adenosine metabolism, resulting in a decrease in the number of CD39 + and CD73 + cells at the maternal–fetal interface. This inhibits trophoblast proliferation and invasion and reduces apoptosis and increases toxicity of dNK cells, which in turn leads to RSA. *LA* lactate, *MCT* the monocarboxylate transporter protein, *AZD3965* MCT-1 inhibitor, *HIF-1α* the hypoxia-inducible factor 1α, *ROS* reactive oxygen species, *SRC* Proto-oncogene tyrosine-protein kinase SRC, *LDHA* lactate dehydrogenase A, *INOS* inducible nitric oxide synthase, *EVT* Extravillous trophoblasts, *dNK* decidual natural killer cells, *TGF-β* transforming growth factor-β, *mTOR* mammalian target of rapamycin, *HGF* hepatocyte growth factor

In conclusion, reduced GLUT protein expression during pregnancy affects glycogen transport synthesis in the endometrium, which leads to insufficient substrates for gluconeogenesis and induces RSA. After reviewing points ii and iii, we found that HIF, as an important factor regulating glucose metabolism homeostasis in the special hypoxic environment of pregnancy, can also be regulated by glucose metabolites, thus leading to miscarriages (details in Fig. 1). HIF activation by LA can promote macrophage M1 polarization to disrupt immune tolerance, leading to miscarriages (details in Fig. 1A); decreased succinate expression promotes the degradation of HIF-1α, which in turn inhibits angiogenesis, trophoblast invasion and migration and glycolysis (details in Fig. 1B); the downregulation of the TGF-β/mTOR pathway reduces HIF-1α expression, which inhibits ATP-adenosine metabolism and increases dNK cell toxicity (details in Fig. 1C).

### Abnormal lipid metabolism and miscarriage

Several studies have shown that abnormal lipid metabolism is associated with spontaneous abortion and pregnancy complications such as endothelial injury, preeclampsia, and gestational hypertension [67–69]. In this section, we detail the studies related to abnormal lipid metabolism and abortion through three aspects: (i) peroxisome proliferator-activated receptors (PPARs); (ii) total polyunsaturated fatty acids (PUFAs), TG and inflammation; and (iii) the arachidonic acid metabolic pathway, leptin and myometrial contractions.

PPARs (PPAR-α, PPAR-β/δ, and PPAR-γ) are members of the nuclear receptor superfamily, acting as ligand-inducible transcription factors and playing crucial roles in glucose and lipid metabolism [70]. Studies have shown that PPAR deficiency inhibits fatty acid uptake and expression of fatty acid transporter proteins and promotes the production and secretion of proinflammatory cytokines, leading to impaired placental development and functional impairment [71–73]. This might account for the high abortion rate in PPARα knockout mice in animal experiments and the fact that mutations in PPARγ and PPARδ cause infertility [74–76]. In addition, a study has demonstrated that PPAR-agonists have antidiabetogenic, anti-inflammatory, and antioxidant effects, which are all potentially beneficial in the treatment of gestational diabetes mellitus (GDM) [77]. This suggests another possible mechanism of action for improvement of pregnancy outcomes. However, determining whether PPAR gene polymorphism is relevant to the development of GDM still requires further evidence [78].

PUFAs, especially n-6 fatty acids, contribute to lipid peroxidation fragility and the proinflammatory effects of the corresponding peroxidation products, which in turn increase oxidative stress, alter lipid metabolism, and disrupt hormones [79–81]. This would lead to lower probability of pregnancy and live birth and increased risk of miscarriage [82]. In addition, it has been proposed that patients with insulin resistance (IR) have significantly higher triglyceride (TG) levels (which brings about an increased ratio of CD3^+^CD4^+^) and numbers of CD3^+^CD8^+^ lymphocytes, reduced insulin sensitivity, and induction of metabolic inflammation, resulting in RSA [83].

Metabolomic analysis revealed that the expression of the arachidonic acid metabolic pathway-related genes cyclooxygenase-1 (COX-1), cyclooxygenase-2 (COX-2), prostaglandin F2α receptor (PTGFR), and thromboxane A2 receptor (TBXA2R) was significantly increased in RSA patients. Related animal experiments have shown that abnormal expression of COX genes and TBXA2R can cause uterine contraction by regulating the cytoplasmic phospholipase A2α (PLA2α)/COX-2 pathway in endometrial stromal cells and inducing increased prostaglandin synthesis, ultimately leading to RSA [84–86]. All of these findings suggest that modulation of the arachidonic acid metabolic pathway may be a prospective therapeutic strategy to alleviate symptoms in women with RSA. It has also been reported that the adipokine leptin can inhibit spontaneous and oxytocin-induced myometrial contractions by increasing NO and cGMP through stimulation of short-type leptin receptors and activation of the NO pathway in a JAK/STAT-dependent manner [87]. However, this trial demonstrated the inhibitory effect of leptin on uterine contractions only in late pregnancy, and it is not yet known whether it can be used in early pregnancy to reduce the incidence of spontaneous miscarriage.

In conclusion, the abnormal lipid metabolism provoked by the decrease in PPAR and the increases in PUFA and TG can promote inflammation and oxidative stress, which would contribute to miscarriage. In contrast, the arachidonic acid pathway increases prostaglandin synthesis to cause myometrial contractions, leading to miscarriage, which might be ameliorated by pathway modulation and leptin.

### Abnormal amino acid metabolism and miscarriage

**Fig. 2:**
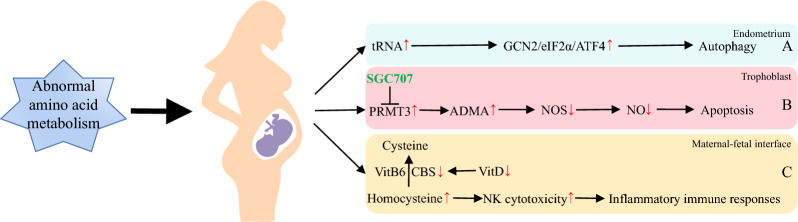
Abnormal amino acid metabolism and miscarriage. **A**. In the endometrium, a decrease in various amino acids can lead to the accumulation of uncharged tRNA, which in turn activates the GCN2/eIF2α/transcriptional activation factor 4 (ATF4) pathway, thereby inhibiting protein synthesis and inducing autophagy. **B**. At the maternal–fetal interface, meconium macrophages can promote trophoblast cell apoptosis by activating the PRMT3/ADMA/NO pathway and decreasing the concentration of NO in the meconium. SGC707 can reverse. **C**. Vitamin D deficiency can increase homocysteine levels by decreasing CBS, while inducing increased NK cell cytotoxicity and promoting inflammatory immune responses at the maternal–fetal interface. *GCN2* the general control nonderepressible 2, *eIF2αK4* eukaryotic translation initiation factor 2α kinase 4, *ATF4* transcription activation factor 4, *PRMTs* type I protein L-arginine methyltransferases, *SGC707* PRMT3 inhibitor, *ADMA* asymmetric dimethylarginine, *NOS* nitric oxide synthase, *NO* nitric oxide, *CBS* cystathionine beta-synthase, *VitB6* Vitamin B6, *VitD* Vitamin D

The regulation of amino acid metabolism in the endometrium is one of the most important metabolic processes to meet the increased nutritional demands of early pregnancy. It provides not only protein components but also intermediate metabolites that promote multiple biosynthetic pathways, which help to establish the metabolic microenvironment of the endometrium in preparation for implantation and early pregnancy [35, 88]. Therefore, we introduce the effects and mechanisms of amino acid metabolism on different tissues during pregnancy from three aspects: (i) autophagy of the endometrium; (ii) apoptosis of trophoblast cells; and (iii) inflammation of the maternal–foetal interface (Fig. 2).

Metabolomic analysis identified 19 decreased metabolites and 22 increased metabolites in the endometrium of women with recurrent miscarriages, including decreases in pyruvate, glutamate and succinate as well as an increase in glutamine [89]. The reduction of various amino acids accounts for the accumulation of uncharged transfer RNA (tRNA), which binds a protein kinase called eukaryotic translation initiation factor 2α kinase 4 (eIF2αK4) and activates the general control nonderepressible 2 (GCN2)/eIF2α/transcription activation factor 4 (ATF4) pathway, thereby inhibiting protein synthesis and inducing autophagy [90]. In addition, glutamine metabolism can induce autophagy through the release of NH3 [91]. This suggests that abnormal collusion between amino acid metabolism and autophagy may contribute to an impaired endometrial microenvironment, which in turn induces RSA (Fig. 2A).

It has been reported that nitric oxide (NO) can inhibit trophoblast apoptosis, thereby reducing adverse pregnancy outcomes [92]. In the placenta, NO is promoted by L-arginine via inducible NOS (iNOS) and endothelial NOS (eNOS) [93]. This process can be selectively inhibited by asymmetric dimethylarginine (ADMA), which originates from type I protein L-arginine methyltransferase (PRMT)-mediated protein degradation [94, 95]. A mechanistic study on the effect of decidual macrophages (DMs) on the regulation of trophoblast apoptosis showed that DM can promote trophoblast apoptosis by activating the PRMT3/ADMA/NO pathway, which reduces the concentration of NO in metaphase and leads to RSA [96]. Furthermore, animal experiments have also revealed that the PRMT3 inhibitor SGC707 could significantly reduce the embryo uptake rate in a mouse model prone to miscarriage, which demonstrates that the PRMT3/ADMA/NO pathway could be a potential target for the treatment of miscarriage. However, clinical and safety trials still need to be completed (Fig. 2B).

Homocysteine, a thiol-containing amino acid, is involved in sulfation and methylation metabolic pathways. Studies have shown that elevated homocysteine levels can activate proinflammatory pathways through endothelial dysfunction and lead to leukocyte-endothelial cell interactions and leukocyte recruitment, causing vascular inflammatory changes that contribute to microembolism at the maternal–foetal interface, which ultimately results in RSA [97–99]. In addition, the trans-sulfuration pathway of homocysteine metabolism requires cystathionine beta-synthase (CBS) and the cofactor vitamin B6. Since the CBS gene is a target of the vitamin D receptor, vitamin D deficiency can increase homocysteine levels by decreasing CBS, inducing increased NK cell cytotoxicity, which in turn further promotes the inflammatory immune response at the maternal–foetal interface and leads to RSA [100–102]. This demonstrated the relationship between amino acid metabolism and inflammation in pregnancy and suggested the necessity of vitamin D and B6 supplementation during pregnancy in RSA patients (Fig. 2C).

In conclusion, abnormal amino acid metabolism can act in several pregnancy-related tissues, leading to adverse pregnancy outcomes. Abnormal amino acid metabolism in the endometrium can induce autophagy leading to an impaired microenvironment; at the maternal–foetal interface, it can be regulated by DM to activate the PRMT3/ADMA/NO pathway and promote trophoblast apoptosis; and increased homocysteine levels at the maternal–foetal interface can induce the inflammatory immune response, leading to abortion, which can be alleviated by vitamin D supplementation.

### Abnormal oxidation–reduction balance and miscarriage

Research on hyperandrogenemia and insulin resistance-related miscarriage in women showed that the important factors contributing to embryonic damage in polycystic ovary syndrome (PCOS)-like conditions include excessive production of ROS, mitochondrial dysfunction, and the inhibition of superoxide dismutase 1 (SOD1) and Keap1/Nrf2 antioxidant responses in the placenta [103]. This suggests that the disturbance in the balance between oxidative stress (ROS production) and antioxidants is responsible for the initiation and development of pathological processes affecting female reproduction [104, 105]. Therefore, we elaborate on the mechanisms by which dysregulation of redox reaction balance contributes to abortion in terms of abnormalities in both oxidative stress and antioxidants.

It has been reported that increased levels of malondialdehyde and lipid peroxides in placental tissue increase ROS, which can lead to sudden and premature formation of maternal placental perfusion while damaging the trophectoderm, resulting in RSA [106, 107]. In addition, activation of the Fas/FasL signalling pathway in villi tissue can promote oxidative stress-induced apoptosis of trophoblast cells, contributing to miscarriage. The molecular mechanism is associated with inhibition of the Notch1 signalling pathway and upregulation of epithelial cadherin (E-cadherin), soluble vascular endothelial growth factor receptor 1 (sFlt-1), and vascular endothelial growth factor (VEGF) expression [108]. It follows that excessive activation of oxidative stress can lead to premature placental perfusion, induction of apoptosis, and destruction of the trophectoderm, which would result in miscarriage.

Changes in the consumption of antioxidants can also lead to disturbances in the balance of pro-oxidant and antioxidant factors, which could lead to miscarriage [109, 110]. Glutathione and glutathione peroxidase are antioxidants that neutralize free radicals and lipid peroxides to maintain intracellular homeostasis and redox balance. In a large case‒control study on genetic polymorphisms of the glutathione family enzyme glutathione S-transferase (GST), an elevated risk of RSA was found to be associated with increased oxidative stress due to null polymorphisms of the GSTM1 and GSTT1 genotypes in RSA patients [111–113]. Nonetheless, epidemiological studies and related experiments have shown that sulfur dioxide (SO2) and its derivatives can inhibit trophoblast cell viability and the ROS/IL-6/STAT3 pathway, interfere with cell proliferation by blocking the cell cycle, induce apoptosis, disrupt the secretion of inflammation-related cytokines, and inhibit cell invasion and migration, leading to miscarriage and pregnancy complications [114]. This reflects the fact that both reduced and inappropriate use of antioxidants can lead to adverse pregnancy outcomes.

Although it is controversial whether antioxidant supplementation could change pregnancy outcomes [115–117], some new findings on antioxidant drugs have been achieved. A study in 2020 revealed that astaxanthin significantly alleviated poor glucose tolerance and beta-cell insufficiency and improved pregnancy outcomes by restoring the Nrf2/heme oxygenase-1 (HO-1) antioxidant pathway in the livers of gestational diabetic mice, inhibiting oxidative stress in vivo, and enhancing the activity of antioxidant enzymes [118]. It has also been reported that alpha lipoic acid (ALA) and its reduced form dihydrolipoic acid (DHLA) may improve pregnancy outcomes through specific stimulatory activity on Nrf2-dependent gene transcription and by the inhibition of NF-kB activity [119, 120], but more patient samples and further studies on safety in pregnancy and the pharmacokinetics of the vaginal pathway are still needed.

## Metabolism-related genes and miscarriage

RSA is considered idiopathic in approximately 50% of cases, thus highlighting the potential genetic and epigenetic origins of the disease [121–123]. While we previously discussed the effects of glucose metabolism, lipid metabolism, amino acid metabolism, and redox reactions on pregnancy outcome, we then attempted to summarize the metabolism-related genes that cause miscarriage to support its heritability (details in Table 1).

**Table 1:** Metabolism-related genes and miscarriage

Gene	Target	Study model	Effect	Refs
SGLT1	SGLT1	C57BL/6 J mice Human Endometrial Stromal Cells	Reduce glycogen synthesis	[52]
PPARα	PPARs	C57BL/6 J mice	Inhibit fatty acid uptake and fatty acid transporter protein expression	[74]
COXs TBXA2R	PLA2α/COX-2	CD1 mice	Increase synthesis of prostaglandins, leading to uterine contractions	[84, 85]
GSTM1 GSTT1	Glutathione	Human blood samples	Increase oxidative stress	[112, 113]
NOS3	NO	Human blood samples	Decrease levels of NO cause vasoconstriction, which in turn leads to impaired placental perfusion and an increased risk of infarction	[124]
EMP2	FAK/Src/HIF-1α	C57BL/6 mice Human placenta samples	Inhibite angiogenesis and oxidative phosphorylation	[127]
ADIPOQ	Lipocalin	Human blood samples	Unknown	[129]
CFHrs1065489G > T	C3	Human blood samples	Homocysteine and prolactin levels	[131]
CFH rs1061170 TC	C3	Human blood samples	Uric acid and triglyceride levels	

*SGLT1* sodium-glucose co-transporter 1, *PPAR* Peroxisome proliferator activated receptor, *COX* cyclooxygenase, *TBXA2R* thromboxane A2 receptor, *PLA2α* phospholipase A2α, *GST* glutathione S-transferase, *NOS3* nitric oxide synthase 3, *EMP2* epithelial membrane protein 2, *FAK* focal adhesion kinase, *Src* steroid receptor coactivator, *ADIPOQ* Adiponectin

An Austrian study linked unexplained miscarriages with a variant of a specific gene called nitric oxide synthase 3 (NOS3) [124]. The data suggest that heterozygous carriers of the NOS3 polymorphism have a 1.6-fold increased risk of RSA, which might be due to reduced levels of NO causing vasoconstriction, which in turn leads to increased risk of impaired placental perfusion and infarction. In addition, another study showed that genetic defects in epithelial membrane protein 2 (EMP2) can inhibit angiogenesis and oxidative phosphorylation by suppressing FAK and Src to inhibit the production of HIF-1α in the trophectoderm, leading to miscarriage [125–127]. The increased recruitment of HIF-1α in NK cells in the uterus of EMP2-/- mice might represent a compensatory mechanism.

Lipocalin is a hormone involved in the regulation of energy, lipid and glucose metabolism and is encoded by the ADIPOQ gene. A study in 2021 demonstrated the contribution of ADIPOQ gene variants to inherited susceptibility to RSA [128]. Of the 14 single nucleotide polymorphisms (SNPs) tested, RSA risk was moderately associated with rs4632532, rs7649121, and rs1501299 and strongly associated with rs17366568, rs2241766, and rs2241767 [129].

Pregnancy can induce complex immune responses at the implantation site to promote and protect the pregnancy. Therefore, immune dysfunction is also considered to be an important cause of spontaneous abortion [130]. The complement system is essential for stable placental and foetal development. It has been verified that polymorphisms of complement factors D (CFD) and H (CFH) can influence pregnancy outcomes through the regulation of C3 [131–133]. Clinical data showed that women with RSA and CFH rs1065489TT genotypes had significantly lower homocysteine levels than women with RSA and CFH rs1065489GG and GT genotypes. In addition, patients with the CFH rs1065489TT genotype had higher prolactin levels than patients with the CFH rs1065489GG and GT genotypes. Patients with the CFH rs1061170TC genotype had significantly higher uric acid and triglyceride levels than patients with the CFH rs1061170TT genotype. Evidence has suggested that the CFH rs1065489G > T polymorphism is related to homocysteine and prolactin levels, and the CFH rs1061170 TC genotype is related to uric acid and triglyceride levels in RSA patients. These results indicated that the complement system could impact pregnancy outcomes through the modulation of metabolism.

In addition, many studies have also shown that the regulation of mitochondrial energy metabolism by nucleic acids such as mtDNA, miR-210, miR-218, miR-574-5p and miR-3135b could lead to pregnancy complications such as foetal growth restriction (FGR), preeclampsia (PE) and GDM [134–137]. However, their roles in spontaneous abortion are relatively unexplored. The link between genetics and metabolism might contribute to further insights into the genetic mechanisms leading to spontaneous miscarriages.

## Conclusion

During pregnancy, the mother undergoes many metabolic adaptations to meet the demands of pregnancy. These adaptations help prepare the mother for breastfeeding and provide the proper environment for normal foetal growth in the uterus. Metabolomic analysis has identified abnormal metabolic indicators in both human miscarriage patients and animal miscarriage models. Studies have revealed that abnormal glucose metabolism, lipid metabolism, amino acid metabolism, and oxidation‒reduction balance can lead to adverse pregnancy outcomes by inducing maternal inflammatory responses, promoting uterine contraction, disrupting immune tolerance, inducing autophagy, activating apoptosis, and inhibiting invasive migration and angiogenesis. In addition, animal studies have been conducted on some of these targets and have demonstrated that drugs targeting metabolic abnormalities can improve pregnancy outcomes in miscarriage models, but further studies and clinical trials are needed to clarify their therapeutic efficacy and safety in women with miscarriage. In this review, we summarize the pathways and related therapeutic agents regarding abnormal metabolism triggering miscarriage (Table 2). This might provide directions for future research and new therapies related to miscarriage.

**Table 2:** Metabolism-related medications

Medication	Target	Study model	Effect	Refs
AZD3965	MCT-1	Male BALB/c, male DBA/2 and female CBA/J mice	Block lactic acid intake	[58]
Leptin	NO/cGMP/PK-G	Swiss albino mice	Inhibit uterine contractions	[87]
SGC707	PRMT3/ADMA/NO	Female CBA/J, male DBA/2 and male BALB/c mice	Inhibit the apoptosis of trophoblast cells	[96]
Vitamin D	CBS	Human blood samples Human placenta samples	Inhibit inflammatory immune response	[102]
Astaxanthin	Nrf2/HO-1	C57BL/KsJ db/ + mice	Inhibits oxidative stress in the body and enhances antioxidant enzyme activity	[118]
ALA DHLA	FAK/Src/HIF-1α	Human samples	stimulate Nrf2-dependent gene transcription and inhibit NF-kB activity	[120]

*MCT-1* monocarboxylate transporter protein 1, *PK-G* protein kinase G, *PRMT3* protein l-arginine methyltransferase 3, *ADMA* asymmetric dimethylarginine, *CBS* cystathionine beta-synthase, *Nrf2* Nuclear factor-erythroid 2 related factor 2, *HO-1* Heme Oxygenase-1, *ALA* alpha lipoic acid, *DHLA* dihydrolipoic acid, *FAK* focal adhesion kinase, *Src* steroid receptor coactivator, *HIF-1α* Hypoxia inducible factor 1 α

## Data Availability

Not applicable.
